# Reaction time-based Concealed Information Test in eyewitness identification is moderated by picture similarity but not eyewitness cooperation

**DOI:** 10.1007/s00426-018-1139-8

**Published:** 2019-01-11

**Authors:** Katerina Georgiadou, Agatha Chronos, Bruno Verschuere, Melanie Sauerland

**Affiliations:** 1 Department of Clinical Psychological Science, Faculty of Psychology and Neuroscience, Maastricht University, P.O. Box 616, 6200 MD Maastricht, The Netherlands; 2 Department of Clinical Psychology, University of Amsterdam, Amsterdam, The Netherlands; 3 Department of Social Psychology, Vrije Universiteit Amsterdam, Amsterdam, The Netherlands

## Abstract

The reaction time-based Concealed Information Test (RT-CIT) has high validity in assessing recognition of critical information. Findings on its usefulness for diagnosing face recognition in eyewitnesses are inconsistent. Experiment 1 (*N* = 82) tested whether closely matching the faces of the probes and irrelevants, as required for a fair lineup, undermines RT-CIT usefulness. Preregistered Experiments 2a and 2b (*N*s = 48), tested the role of eyewitness cooperativeness for RT-CIT validity. All participants watched a mock crime video and then completed an RT-CIT. As expected, the usefulness of the RT-CIT was moderated by picture similarity, with better detection for non-matched faces. Unexpectedly, eyewitness cooperation (conceal vs. reveal recognition), did not affect the validity of the RT-CIT. A large CIT effect observed in Experiment 2b further suggested that—even with matched faces—the RT-CIT might be of use when encoding conditions during the crime were favorable. Cases in which witnesses are unwilling or afraid to make an explicit identification might concern another possible application.

## Introduction

Eyewitness identifications have a prominent impact on police investigations and criminal proceedings. They can help resolve a case (Davis, Jensen, Burgette, & Burnett, 2014), but can also contribute to wrongful convictions (http://www.innocenceproject.org; National Research Council, 2014). Meta-analyses show that the average decision accuracy for 6-person lineups revolves around 50% (e.g., Clark, Howell, & Davey, 2008; Fitzgerald & Price, 2015; Steblay, Dysart, & Wells, 2011). While proper lineup construction and administration can increase accuracy rates (e.g., Brewer & Palmer, 2010), scholars have recently voiced a need for alternative identification procedures (Brewer & Wells, 2011; Wells, Memon, & Penrod, 2006). Here, we will test the usefulness of one such potential alternative, namely the Concealed Information Test (CIT; Lykken, 1959; Verschuere, Ben-Shakhar, & Meijer, 2011).

The CIT assesses the recognition of information. In criminal cases, it does so by presenting suspects with multiple options related to a key feature of the crime (such as the weapon or location) and assessing their reactions to each option using physiological and behavioral measures (e.g., skin conductance, respiration line length, event related potentials, reaction times). The reaction time-based CIT (RT-CIT; Seymour, Seifert, Shafto, & Mosmann, 2000) commonly entails the sequential, rapid presentation of crime-related (i.e., probe) and unrelated (i.e., irrelevants) options on a computer. For instance, if a homicide is investigated in which the murder weapon used was a kitchen knife, stimuli in the RT-CIT may be a picture of the kitchen knife and of other possible murder weapons (e.g., a hammer, a baseball bat, a pistol, a switchblade). Examinees are required to respond to all items by pressing one of two buttons. To ensure that they process the stimuli, examinees respond with a dedicated button for a set of stimuli (called targets) memorized just before starting the CIT and with another button for all other stimuli. In the example above, the examinee may be asked to press one button for the target hammer, while pressing a different button for all the other stimuli. The core idea behind the RT-CIT is that only an individual with knowledge of the crime will respond differently to the crime-related stimuli (i.e., probes) as compared to the irrelevant stimuli.

The RT-CIT has been used in many experiments to assess the recognition of objects and verbal stimuli. A meta-analysis of 34 studies with 1063 participants in total reported a large RT-CIT effect (Cohen’s *d* = 1.30; Suchotzki, Verschuere, Van Bockstaele, Ben-Shakhar, & Crombez, 2017). Its diagnostic value (expressed as area under the curve) has been found to be 0.82 (Meijer, Verschuere, Gamer, Merckelbach, & Ben-Shakhar, 2016). This means that there is an 82% probability that a person picked at random from those who recognize the stimulus (i.e., someone who possesses crime-related information) responds more distinctively than a person picked at random from those who do not recognize the stimulus (i.e., do not possess crime-related knowledge).

The RT-CIT is also capable of detecting face recognition in participants who thoroughly memorized previously unseen faces just prior to taking the CIT (Seymour & Kerlin, 2008). Furthermore, the RT-CIT has shown to be particularly useful for highly familiar (e.g., sibling) faces when participants concealed recognition (by pressing the ‘do not recognize’ button), but not for less familiar faces (e.g., teacher) when participants were revealing recognition (Meijer, Smulders, Merckelbach, & Wolf, 2007). This suggests that face familiarity, individually or in combination with intent to deceive may be critical for assessing face recognition in the RT-CIT.

Further exploring the usefulness of the CIT as an alternative identification procedure, Sauerland, Wolfs, Crans, and Verschuere (2017) tested the utility of the RT-CIT in a typical eyewitness setup. Across five experiments, participants viewed a video of a mock crime before taking the RT-CIT. The pictures included in the CIT were carefully matched in terms of physical characteristics following the guidelines of explicit eyewitness identification procedures (Wells et al., 1998). A mini meta-analysis across these five experiments revealed a negligibly small effect size of *d* = 0.14. It is the aim of the current work to test two possible explanations for this unexpectedly small effect size. First, the physical similarity of the faces (i.e., matched faces) required in a fair lineup may have lowered the diagnosticity of the RT-CIT (Sauerland et al., 2017). Note that the facial images used in previous studies (Meijer et al., 2007; Seymour & Kerlin, 2008) were not similar according to physical characteristics (i.e., non-matched). If the RT-CIT is to meet eyewitness identification guidelines (Technical Working Group for Eyewitness Evidence, 1999; Wells et al., 1998), the physical characteristics of the distractors should match those of the suspect (or of the perpetrator description) to minimize the possibility of a wrongful identification (Brewer & Palmer, 2010; Fitzgerald, Price, Oriet, & Charman, 2013). There is indeed evidence to suggest that the size of the CIT effect varies with the similarity between the probe and the irrelevant stimuli (Ben-Shakhar & Gati, 1987).

A second explanation for the limited usefulness of the RT-CIT as an identification procedure in Sauerland et al. (2017) includes the possibility that the intent to deceive is critical for the RT-CIT to be beneficial (see Meijer et al., 2007). Indeed, when instructing participants to either conceal or reveal their knowledge regarding an event, deception was found to be critical for a large RT-CIT effect to occur (Suchotzki, Verschuere, Peth, Crombez, & Gamer, 2015). Thus, the RT-CIT effect is not merely driven by recognition, but also by a response conflict and attempting response inhibition when deceiving (for further CIT studies manipulating cooperativeness see klein Selle, Verschuere, Kindt, Meijer, & Ben-Shakhar, 2015, 2017; Zvi, Nachson, & Elaad, 2012).

## Overview of experiments

In Experiment 1, we manipulated stimulus similarity by exposing participants to either matched or non-matched picture versions of the RT-CIT after watching a mock crime. The matched condition constitutes a direct replication of Sauerland et al. (2017, Experiment 4). In the non-matched condition, the physical characteristics of the presented faces displayed greater diversity than in the matched condition. We expected the RT-CIT to be more useful for diagnosing face recognition for non-matched pictures, compared to matched ones.

Experiments 2a and 2b tested whether the validity of the RT-CIT in eyewitness identifications depends on response inhibition due to deliberate concealment. The setup for the cooperative condition of Experiments 2a and 2b was similar to Sauerland et al. (2017, Experiment 4). For the uncooperative condition, we instructed participants to conceal recognition (see klein Selle et al., 2015, 2017; Suchotzki et al., 2015, for a similar approach). We expected the RT-CIT to be more useful for uncooperative vs. cooperative witnesses. Because post hoc exploratory analyses of Experiment 2a suggested the role of response inhibition would only manifest when explicit recognition was given, Experiment 2b maximized explicit recognition by increasing exposure time to the probes.

## Experiment 1

### Method

#### Participants

Based on Sauerland et al. (2017, Experiment 4), we aimed for about 80 participants. Ninety-four participants were tested (77 females; *M*_age_ = 21.88, SD_age_ = 4.19). Twelve participants were excluded because they knew one or more of the individuals presented in the RT-CIT personally. No participants were excluded based on high error rates (i.e., ≥ 50%) for any response category (i.e., responses to probes, targets, or irrelevants) or a large number of non-completed trials (i.e., ≥ 50%; cf. Kleinberg & Verschuere, 2016). The remaining 82 participants were 66 females and 16 males aged 18–43 years (*M*_age_ = 21.83, SD_age_ = 4.32). Participants’ native languages were German (52.4%), Dutch (23.2%), English (8.5%), French (3.7%), Greek (2.4%), or other (9.8%). The majority were students (95.1%) while the rest were working professionals. Participants could choose between either study credit (1/2) or a €5 gift voucher for their participation. The study was approved by the Ethics Committee of the faculty.

#### Design

Participants were randomly assigned to the matched faces (*n* = 42) or non-matched faces (*n* = 40) conditions. A mixed 2 (stimulus similarity: matched vs. non-matched) × 2 (stimulus type: probes vs. irrelevants) factorial design was used to assess the effect of face matching on reaction times for different stimulus types.

#### Materials

##### Stimulus video

The stimulus video used in all three experiments was taken from Sauerland et al. (2017, Experiment 4). The short clip of approximate duration 1:13 min involved a cell phone theft. One of two female actors seizes the opportunity to steal the other actors’ phone after an accidental encounter. The clip existed in two versions in which the two actors’ roles as thief and victim were interchanged. A detailed description can be found in Sauerland et al. (2017).

##### Selection and preparation of facial stimuli

On all photographs, individuals wore their hair loose, did not wear accessories, jewelry, or glasses, and displayed a neutral facial expression. Photographs were taken against a white wall and cropped in such a way as to depict each individual from the collarbone up. Two sets of stimuli were created, namely the matched and the non-matched pictures.

The matched pictures were the same as the ones used by Sauerland et al. (2017, Experiment 4) and consisted of 2 × 6 photos of different individuals. They were selected to match the physical description of each of the two probes (i.e., actresses in the film) using a standard procedure for selecting pictures for a fair six-person photo lineup (Doob & Kirshenbaum, 1973; Malpass & Lindsay, 1999; Tredoux, 1999). Specifically, in pilot work, independent groups of mock witnesses (*N*s varying between 23 and 31) were presented with a short description of the actor and then presented with a set of six pictures that either included the probe or did not include the probe. Tredoux’s *E* (i.e., the effective lineup size) ranged from 3.8 to 4.8 (of a possible 6), thereby marking them as a fair picture selection (Tredoux, 1998, 1999). One of the six pictures was randomly selected to serve as target for each participant, while the remaining five pictures served as irrelevants in the CIT protocol.

The non-matched pictures were selected to differ in hair color, length, texture, eye color, gender, and age from the referring probe. These pictures were selected so that no single person would stand out in comparison with the rest (e.g., carrying unique physical characteristics; Wells et al., 1998); which was ascertained by a pilot study completed before the experiment took place. The results of the pilot study revealed no significant differences in the reaction times for any of the presented pictures, therefore indicating that no picture was salient enough to elicit a differential reaction time. We are unable to present the stimuli here as we do not have consent from the individuals depicted to do so.

##### Reaction time-based Concealed Information Test

The RT-CIT protocol used across experiments was adapted from Sauerland et al. (2017, Experiment 4). The RT-CIT was programmed using Presentation software (version 20.0), and the facial stimuli presented were approximately 220 × 260 pixels in size. In short, participants were asked to memorize two facial pictures that were presented for 30 s at the beginning of the task (i.e., the targets) for which they had to respond by pressing a specific button labeled as YES on the screen. Their response for all other pictures should be the button labeled as NO. Each picture was shown sequentially with the caption ‘Do you recognize this?’ above it, with the YES and NO options presented on the top left and right sides of each picture (locations were counterbalanced). Stimuli were presented for a maximum of 1500 ms, that is, a response for each picture had to be made as fast as possible with a time limit of 1500 ms. Once a participant responded to a picture, the next one appeared; that is, no fixed interstimulus interval was present and the participant had control over the speed of the subsequent stimuli presented.

Participants completed a practice block and received feedback marking their responses as “correct”, “wrong”, or “too slow” if they took longer than 1500 ms to respond. To complete the practice block successfully, participants had to respond correctly for at least 50% of the target pictures. If the correct response rate was lower, additional practice blocks had to be completed until the minimum correct response rate was achieved. Upon successful completion of the practice block, the pictures of the two targets would be presented once again for 15 s. The main task then commenced, being identical to the practice block, except that no feedback was given.

The facial stimuli presented in the task consisted of two probes (i.e., the two actors from the film), two randomly selected targets (i.e., the two faces participants encoded at the beginning of the CIT), and ten irrelevants (i.e., faces that were neither seen in the film nor encoded). During the practice block, each picture was presented twice, resulting in 28 trials. During the main task, each picture was displayed 21 times, resulting in a total of 294 trials per condition.

##### Photo display

To test participants’ explicit recognition of the two actors, participants were presented with a photo display that depicted the facial images of the actors, targets and irrelevants. It consisted of 14 photos arranged in four rows of four or three photos. Each image was numbered (1, 2, 3, etc.).

#### Procedure

Participants completed the informed consent form and provided demographics. They were then requested to remove any possible distractions they had on them (i.e., switching off/muting personal mobile phones). Before participants started watching one of the two stimulus film versions, they were instructed to observe carefully and pay special attention to the faces of the individuals involved. Subsequently, the RT-CIT started. The experimenter remained with the participants for the practice block to answer any questions, and left the lab when the main task started. Next, participants were asked whether they recognized any of the people presented in the pictures from outside of the experiment (i.e., from everyday life). Participants were then handed the photo display depicting the faces of all individuals included in the CIT and were asked to indicate the two actors from the film. If participants were unsure, they were prompted to give their best guess (i.e., forced choice). Finally, participants were debriefed, reimbursed and thanked for participating. The whole experiment lasted about 15–20 min.

### Results and discussion

All data are available on the open science framework (https://osf.io/syr3u/).

#### Data preparation and overview of analyses

Only correct trials (i.e., excluding trials with behavioral errors such as pressing YES for the probes and irrelevants or NO for the targets) with a reaction time between 150 and 1500 ms were taken into account.^1^ Next, reaction times were aggregated to result in mean reaction times per stimulus. These mean reaction times were then further grouped to form variables for the different stimulus types (e.g., probes, targets, and irrelevants). To compare the reaction times for the different stimulus types between matched and non-matched pictures, a mixed measures ANOVA was conducted with stimulus type and matching condition as factors.^2^ Post-hoc comparisons were conducted using paired sample *t* tests.

#### Reaction time comparison between probes and irrelevants

**Fig. 1:**
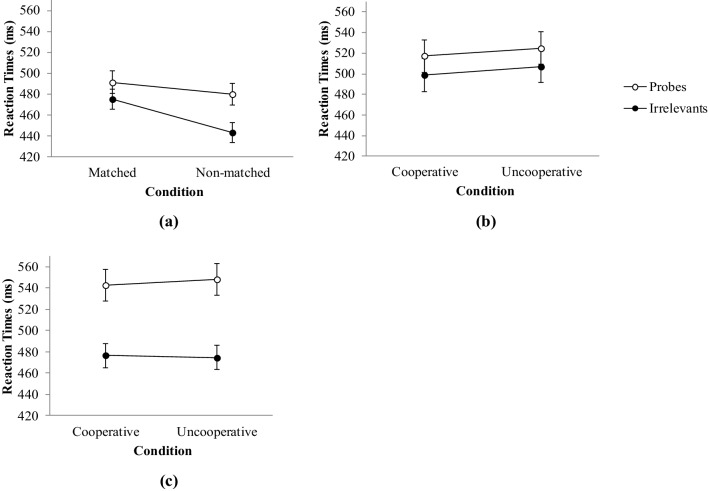
Average reaction times of probes and irrelevants (in ms with error bars displaying 95% CI) for matched vs. non-matched picture conditions in Experiment 1 in **a**, and for cooperative vs. uncooperative participants in Experiment 2a and 2b (**b, c**), respectively

Table 1 offers an overview of the descriptive and inferential statistics. A significant main effect of stimulus type, *F*(1, 80) = 44.44, *p* < .001, *η*^2^ = 0.36, but not matching, *F*(1, 80) = 2.47, *p* = .120, *η*^*2*^ = 0.03, emerged. The expected stimulus type × matching interaction was statistically significant, *F*(1, 80) = 7.14, *p* = .009, *η*^*2*^ = 0.08. Post-hoc comparisons showed that the difference between probes and irrelevants was statistically significant for both matched pictures, *t*(41) = 3.22, *p* = .003, *d* = 0.50, 95% CI [0.11, 0.88], and non-matched pictures, *t*(39) = 5.90, *p* < .001, *d* = 0.93, 95% CI [0.51, 1.36], with the effect being stronger in the non-matched condition. Figure 1a displays this interaction effect.

**Table 1:** Descriptive and inferential statistics for pairwise comparisons of the reaction times (ms) for probes and irrelevant stimuli (including reaction times 150–1500 ms)

Study	Pictures	Motivation	Stimuli	*df*	*t*	*d*	*p*	Mean response time in ms (SD)	
								Probes	Irrelevants
Experiment 4 (Sauerland et al., 2017)	Matched	Cooperative	Thief	74	2.48	0.29	.015	479 (64)	466 (51)
			Victim	74	2.12	0.25	.037	479 (61)	469 (55)
			Across roles	74	3.37	0.39	< .001	479 (55)	467 (51)
Experiment 1	Matched	Cooperative	Thief	41	1.96	0.30	.057	490 (76)	476 (55)
			Victim	41	2.99	0.46	.005	492 (70)	474 (58)
			Across roles	41	3.22	0.50	.003	491 (68)	475 (55)
	Non-matched	Cooperative	Thief	39	5.61	0.89	< .001	496 (85)	443 (65)
			Victim	39	2.75	0.43	.009	464 (70)	444 (64)
			Across roles	39	5.90	0.93	< .001	480 (70)	444 (63)
Experiment 2a	Matched	Cooperative	Thief	23	1.55	0.32	.135	515 (77)	498 (61)
			Victim	23	2.43	0.49	.023	519 (71)	500 (66)
			Across roles	23	2.94	0.60	.007	517 (64)	499 (63)
	Matched	Uncooperative	Thief	23	1.89	0.39	.070	513 (93)	500 (92)
			Victim	23	2.44	0.49	.023	523 (109)	501 (95)
			Across roles	23	3.32	0.67	.003	525 (93)	507 (90)
	Across conditions and roles			47	4.44	0.64	< .001	521 (79)	503 (77)
Experiment 2b	Matched	Cooperative	Thief	23	5.03	1.03	< .001	537 (82)	468 (53)
			Victim	23	5.18	1.06	< .001	548 (92)	484 (59)
			Across roles	23	5.94	1.21	< .001	542 (79)	476 (55)
	Matched	Uncooperative	Thief	23	5.55	1.13	< .001	553 (71)	478 (59)
			Victim	23	5.09	1.04	< .001	545 (76)	471 (55)
			Across roles	23	5.80	1.18	< .001	548 (66)	474 (56)
	Across conditions and roles			47	8.35	1.21	< .001	545 (72)	475 (55)

#### Actor recognition from photo display

A binomial test against 1/7 odds (chance level of 0.15) showed that participants identified the thief (*M* = 0.85) and the victim (*M* = 0.90) above chance level, *p* < .001.^3^

Experiment 1 showed that facial recognition was more successful when using non-matched rather than matched faces in the RT-CIT. This effect can be attributed to faster rejection of irrelevant stimuli in the non-matched vs. the matched condition. These results are in line with our hypothesis. To further explore additional variables that could moderate facial recognition by means of the RT-CIT, two more experiments were conducted in which eyewitness cooperativeness (cooperative vs. non-cooperative) was tested as a possible moderator.

## Experiments 2a and 2b

The preregistration of the sampling plan, variables, methods, experimental script, and analysis plan of Experiment 2a and 2b are available on the open science framework (osf.io/4wksy; osf.io/4y7ge). The following sections apply to both studies. The experiments were approved by the Ethics Committee of the faculty.

### Method

#### Power analysis

To estimate the necessary sample sizes we conducted a power analysis with G*Power (Faul, Erdfelder, Lang, & Buchner, 2007, 2009) for a mixed measures ANOVA, including an interaction. The most relevant study available to base our effect size estimate on was Suchotzki et al. (2015) who observed a large effect (*d* = 0.97) of concealment on the RT-CIT (analogous *f* = 0.49; DeCoster, 2012). Additionally, we used an *α* error probability of .05 and a *β* of .95, with the number of groups and measurements set to 2. The Pearson correlation among repeated measures was taken from Sauerland et al. (2017; *r* = .70) and nonsphericity correction was set to 1. This analysis resulted in a minuscule sample size of only 12 participants (6 per experimental condition). Following rule #2 of Simmons, Nelson, and Simonsohn’s (2011) seminal paper on avoiding false-positives, namely authors must collect at least 20 observations per cell, or else provide a compelling cost-of-data-collection justification (p. 1363), we decided to collect a sample of 48 participants per study, with 24 participants in each between-subjects condition.

#### Participants

For Experiment 2a, 48 participants (36 females; *M*_age_ = 24.71 years, SD_age_ = 8.92) were recruited through advertising on the laboratory web page of the university. None had to be excluded based on our predetermined inclusion and exclusion criteria (cf. Kleinberg & Verschuere, 2016; see Experiment 1). Most participants were students (89.6%) or pursuing professional careers. The most frequent native languages were Dutch (50%), English (12%), German (6%), or other (32%).

For Experiment 2b, 50 participants were recruited, 2 of which were excluded based on revised inclusion and exclusion criteria that were applied in this study. More specifically, we excluded participants who could not explicitly identify the probes from a photo display following the RT-CIT, in addition to the rest of the criteria mentioned above (see Experiment 1). Of the 48 included participants (32 females, *M*_age_ = 22.58, SD_age_ = 4.83), the majority were students (85.4%) while the rest were working professionals. The majority of native languages were Dutch (40%), English (10%), German (8%), Spanish (8%) or other (34%).

Participants in both experiments could choose between either study credit (3/4) or financial reimbursement (€7.50) for their participation and had the chance to receive an additional bonus (1/2 study credit or €5) based on their performance.

#### Design

Participants were randomly assigned to the cooperative (*n* = 24) and non-cooperative (*n* = 24) conditions. A mixed 2 (cooperativeness: cooperative vs. non-cooperative) × 2 (stimulus type: probes vs. irrelevants) factorial design was used to assess the effect of participant cooperation on reaction times for different stimulus types.

#### Materials

##### Reaction time-based Concealed Information Test

The RT-CIT used was largely the same as the one described in Experiment 1, with the following alterations. The RT-CIT was programmed using Inquisit presentation software (version 5) and the facial stimuli measured 208 × 260 pixels. Each picture was shown sequentially with the caption ‘Is this the target?’ (rather than “Do you recognize this?”, as was asked in Experiment 1), and the locations of the YES and NO answers were not counterbalanced, but constant across all trials (NO on the left; YES on the right). After the practice block was completed, the target pictures were shown again for 30 s (not 15 s), and response feedback was given throughout the practice block and the main task. In addition, during the main task, the stimuli were presented with a randomized interstimulus interval of either 250 ms, 500 ms, or 750 ms. Each picture was displayed 30 times, resulting in a total of 420 trials per condition. An additional probe-learning phase was added in Experiment 2b. Here, participants viewed the CIT probe pictures separately (rather than simultaneously) for 30 s with instructions to memorize these faces because these individuals were involved in the crime they were about to witness in the stimulus film. Following the presentation of the stimulus film, the probe pictures were shown again, separately for 30 s each.

##### Facial stimuli

Across both experiments, the matched pictures of Experiment 1 were used.

##### Photo display

The photo display depicted the facial images of the actors, targets and irrelevants. It consisted of 14 photos arranged in two rows of seven photos with a caption under each photo stating “Suspect 1”, “Suspect 2”, and so on.

##### Memory and manipulation check

A manipulation and motivation check was included (“How motivated were you to reveal the identities of the victim and perpetrator?” and “How motivated were you to conceal the identities of the victim and perpetrator?”) as well as questions regarding the film to ensure participants paid attention to the stimulus event and had seen the probes, for example: “Where did the thief and victim bump into each other?”

#### Procedure

Experiments 2a and 2b followed roughly the same procedure as Experiment 1, with a few additions. The only procedural difference between experiments 2a and 2b concerns an additional exclusion criteria and probe-learning phase in Experiment 2b. After watching the stimulus film, participants were informed that the thief from the film was either their friend (who they needed to help avoid being identified by police) or their enemy (who they wanted to help the police identify), by randomly receiving one of the two conditional instructions for (non-)cooperation. In the cooperative condition, participants were told the following:

You have just witnessed a crime and the police are going to interview you as a witness. You realize that the perpetrator in the crime is a long standing enemy of yours and you want to help the police catch them. You decide to reveal everything you know about the crime (the identity of the perpetrator and victim) to the police so that they catch the perpetrator.

In the non-cooperative condition, participants are told the following:

You have just witnessed a crime and the police are going to interview you as a witness. You realize that the perpetrator in the crime is a long standing friend of yours and you want to make sure the police doesn’t catch them. You decide to conceal everything you know about the crime (the identity of the perpetrator and victim) from the police so that they won’t catch the perpetrator.

Participants were then informed that they would receive additional money or study credit if they successfully managed to reveal or conceal their knowledge based on their given instructions. Upon completion of the CIT, participants called the researcher back in who subsequently explained that they “must ignore their previous instructions to cooperate or not and answer honestly during the following part of the experiment.” This would not affect their bonus. This was to ensure that participants in the non-cooperative condition did not believe that they had to continue hiding their recognition during the survey and photo display. The participants received the photo display after the completion of the CIT, as well as a memory and manipulation check. Finally, participants were debriefed and reimbursed. The whole experiment lasted approximately 45 min.

### Results and discussion

All data are available on the open science framework (https://osf.io/syr3u/).

#### Data preparation and overview of analyses

Data were prepared and aggregated as in Experiment 1. To compare the reaction times of the different stimulus types between cooperative and non-cooperative participants, a mixed measures ANOVA was conducted with stimulus type and participant cooperation as factors.

#### Manipulation check

Manipulation checks on participants’ self-reported motivation to conceal or reveal their information deemed the experimental manipulation successful. More specifically, participants in the conceal condition (*M* = 4.33, SD = 0.87) were more motivated to conceal than participants in the reveal condition (*M* = 2.17, SD = 1.47); *t*(46) = 6.24, *p* < .001; *d* = 1.79, CI [1.3, 2.3]. Vice versa, participants in the reveal condition (*M* = 4.50, SD = 0.89) were more motivated to reveal than participants in the conceal condition (*M* = 2.58; SD = 1.61); *t*(46) = 5.10, *p* < .001; *d* = 1.48, CI [1.0, 1.9].

#### Experiment 2a: preregistered analyses

The 2 × 2 repeated measures ANOVA showed a significant main effect of stimulus type, *F*(1, 46) = 19.31, *p* < .001, ${\eta ^2}$ = 0.29, but not cooperation, *F*(1, 46) = 0.13, *p* = .72, ${\eta ^2}$ < 0.01. The expected stimulus type × participant cooperation interaction was statistically non-significant, *F*(1, 46) < 0.01, *p* = .973, ${\eta ^2}$ < 0.01. This means that RTs for probes (*M* = 521.04, SD = 79.21) were longer than RTs for irrelevants (*M* = 503.05, SD = 77.04), *d* = 0.64, 95% CI [− .02, 1.31], but this effect was not moderated by participant cooperation (see Fig. 1b).

#### Experiment 2a: exploratory analyses

##### Actor recognition from photo display

A binomial test against 1/7 odds (0.15 chance level) showed participants identified the thief (*M* = 0.54) and the victim (*M* = 0.52) above chance level, *p* < .001.^4^

##### Reaction time comparison between probes and irrelevants with only correct explicit identifications

Because response inhibition may only be at play when explicit recognition is given (there needs to be recognition to suppress recognition), we conducted a 2 × 2 ANOVA with stimulus type and participant cooperation as factors including only data from participants who successfully identified both the thief and victim during the photo display (*n* = 18). This was done to verify the existence of an effect of condition while controlling for lack of memory. Results again showed a significant main effect of stimulus type, *F*(1, 16) = 6.51, *p* = .021, ${\eta ^2}$ = 0.29, but not participant cooperation, *F*(1, 16) = 0.43, *p* = .523, ${\eta ^2}$ = 0.03. The interaction was statistically significant, *F*(1, 16) = 5.66, *p* = .030, ${\eta ^2}$ = 0.26. Post-hoc comparisons showed that reaction times regarding probes vs. irrelevants differed somewhat for non-cooperative witnesses (*M* = 507.49, SD = 84.06; *M* = 480.14, SD = 67.43), *t*(7) = 2.37, *p* = .050, *d* = 0.68, 95% CI [− 0.89, 2.25]. No such effect was found for cooperative witnesses, *t*(9) = 0.25, *p* = 0.808, *d* = 0.02, 95% CI [− 1.94, 1.98]. This provides preliminary (albeit post-hoc and underpowered) support of the role of cooperation in the RT-CIT.

The outcome of these exploratory analyses prompted us to conduct Experiment 2b, in which we held recognition constant by including only data with correct lineup identifications and increasing participants’ exposure to the probe stimuli.

#### Experiment 2b: preregistered analyses

Results of the 2 × 2 repeated measures ANOVA showed a significant main effect of stimulus type, *F*(1, 46) = 68.56, *p* < .001, ${\eta ^2}$ = 0.59, but not cooperation, *F*(1, 46) = 0.01, *p* = .913, ${\eta ^2}$ < 0.01, and no stimulus type × cooperation interaction, *F*(1, 46) = 0.21, *p* = .649, ${\eta ^2}$ = 0.01. More specifically, RTs for probes (*M* = 545.42, SD = 72.34) were slower than for irrelevants (*M* = 475.48, SD = 55.07), *d* = 1.21, 95% CI [0.52, 1.89] but this effect was not moderated by participant cooperation (see Fig. 1c).

In contrast to our predictions, participant cooperation did not moderate the CIT effect for faces in both Experiments 2a and 2b. Our exploratory analyses on the effect of cooperativeness including only participants with correct explicit identifications produced the expected results. We followed up with Experiment 2b, in which explicit recognition was guaranteed. Even with enhanced explicit identification in Experiment 2b, cooperation did not moderate the CIT effect.

## General discussion

While the validity of the RT-CIT has been thoroughly established (for meta-analyses see Meijer et al., 2016; Suchotzki et al., 2017; for theoretical analysis see Verschuere et al., 2011), it appeared to be unsuitable for facial recognition in cooperative eyewitnesses (Sauerland et al., 2017). In the current line of research, we sought to explain this discrepancy, by examining two factors that may render RT-CIT unsuitable as an eyewitness identification procedure: face matching and witness cooperation. The role of face matching was confirmed in Experiment 1, with the RT-CIT effect being stronger for non-matched faces (resembling the typical use of the RT-CIT in previous studies) than for closely matched faces (as is required for eyewitness identification). The role of witness cooperation was not supported by the data, as the intent to conceal vs. reveal face recognition did not moderate the RT-CIT effect (Experiments 2a and 2b).

### Close matching

The basic principle of the CIT is that, for the knowledgeable examinee only, the critical stimulus will stand out in comparison to the irrelevant stimuli. While safeguarding that the stimuli remain homogenous for the unknowledgeable examinee, the examiner will try to maximize the CIT effect by choosing stimuli that are maximally different. Take for instance the case of a theft of 450 €. The CIT may ask about the stolen object by embedding the correct item (cash) among several plausible, yet maximally different alternatives (smartphone, laptop, jewels). Contrasting the stolen amount with highly similar items (440 €, 470 €, 490 €) runs the risk that the knowledgeable examinee may no longer differentiate between them. Indeed, the greater the resemblance between the critical and the control items, the smaller the CIT effect (Ben-Shakhar & Gati, 1987). Wells et al.’s (1998) rule 3 on the construction of lineups and photospreads, however, states that “The suspect should not stand out […] based on the eyewitness’s previous description of the culprit or based on other factors that would draw extra attention to the suspect” (p. 630). While rule 3 implies that close matching of the facial CIT stimuli is required for eyewitness recognition in practice, our current data and CIT theory show that such matching diminishes the validity of the CIT.

### Witness cooperation

Based on the idea that response inhibition was critical for the RT-CIT effect and previous data (Suchotzki et al., 2015), we reasoned that witness cooperation would be an important moderator, such that the RT-CIT would paradoxically be more effective in uncooperative than cooperative witnesses. Our two preregistered studies did not confirm such reasoning. Given the available evidence on the role of response conflict in the RT-CIT (Lukasz et al., 2017; Suchotzki et al., 2015; Verschuere et al., 2011; Verschuere, Kleinberg, & Theocharidou, 2015; Zvi et al., 2012), methodological constraints need to be considered. That is, in hindsight, and despite the successful manipulation check, one may question whether we successfully created conditions that differed in the need for response inhibition. In Experiments 2a and 2b we refrained from asking ‘Do you recognize this?’ because this would lead to different response in cooperative (YES) and non-cooperative conditions (NO), biasing the comparison of conditions (cf. Suchotzki et al., 2015). Nonetheless, participants in both the cooperative and non-cooperative conditions were required to press NO when faced with familiar faces, when asked ‘Is this a target?’ This was intended to induce response inhibition in the non-cooperative participants. However, it may also have created response inhibition in cooperative participants, because the instructions—be cooperative yet still press NO for the familiar faces—were contradictory (klein Selle et al., 2015, 2017).

### Encoding conditions

A notable observation was that the CIT effect size more than doubled from Experiment 2a to 2b. The most important difference from 2a to 2b was that explicit recognition was guaranteed in 2b. This observation aligns with the idea that the validity of the CIT is closely coupled to explicit memory (see also Iacono, Boisvenu, & Fleming, 1984; Waid, Orne, & Orne, 1981).

While the use of non-matched faces is not an option in applied contexts (cf. Wells et al., 1998, rule 3), our findings point to other situations in which the matched face-CIT might be diagnostic. More specifically, we found a large CIT effect size in Experiment 2b using matched faces, when encoding conditions were favorable. Compared to Experiment 2a, a minute of full, undivided attention was added for each of the two probes in Experiment 2b, deeming exposure time ‘long’ (cf. Memon, Hope, & Bull, 2003). Furthermore, during this additional encoding time, the presented stimulus material was identical with the CIT images, mirroring a situation in which a perpetrator’s appearance is very similar during the crime and the identification procedure. The observed increase in effect size from Experiment 2a to 2b can probably be attributed, at least in part, to an optimization of encoding conditions, that is, prolonged exposure time and increased encoding-testing stimulus similarity. If replicated, this would suggest that the CIT might be useful in an applied setting when encoding conditions were favorable. Another possible application might concern cases in which witnesses are uncooperative in the sense that they experience explicit identification, but are unwilling or afraid to verbalize this, because they fear the consequences in the context of organized or gang-related crime (for similar argumentation see Visu-Petra, Jurje, Ciornei, & Visu-Petra, 2016).

## Limitations

The current findings must be considered in light of the limitations of the studies. While we observed a moderate (matched condition Experiment 1: *d* = 0.50; Experiment 2a: *d* = 0.64)-to-strong (non-matched condition Experiment 1: *d* = 0.93; Experiment 2b: *d* = 1.21) RT-CIT effect for faces across three studies, they all used the same stimulus materials (with various procedures). Although the current results suggest that the RT-CIT could be an option in real live cases, good encoding conditions provided, this conclusion is based on a single set of materials, and therefore requires replication. Furthermore, the large effect sizes observed in Experiment 2b concern a sample with 100% accurate explicit recognition. This was facilitated by an additional probe-learning phase, in which the CIT probe picture was presented. Strictly speaking, participants’ task during the CIT protocol thus concerned a picture recognition rather than a face recognition task (Bruce, 1982). While we acknowledge that this lowers ecological validity, this procedure was instrumental in our aim to examine whether inhibition (cooperative vs. non-cooperative eyewitness) played a role for those participants who recognized the thief and victim. The applicability of the RT-CIT as an identification procedure in real cases will depend on whether the usefulness of the tool can be replicated under more realistic, favorable encoding conditions.

## Conclusion

To sum up, while identifying conditions that hinder the RT-CIT for establishing perpetrator identity (close matching), we also identified very specific situations under which it could form a promising alternative to current explicit identification procedures. These include crimes with relatively long exposure time (60 to 90 s), allowing good viewing conditions, close resemblance of the perpetrator at the time of the crime and the identification procedure, and cases in which witnesses do not want to make an explicit identification, for example because they are threatened.

## Footnotes

1 Following previous work, data were also analyzed with a response deadline of 800 ms (Kleinberg & Verschuere, 2015; Noordraven & Verschuere, 2013; Verschuere, Crombez, Degrootte, & Rosseel, 2010) for all experiments. These results are reported in an online appendix on the open science framework (osf.io/4y7ge) to allow possible comparison with studies using the 800 ms deadline. Here, we report the findings with the 1500 ms deadline, because there is reason to believe that processing time for faces is often longer than 800 ms (e.g., Ramon, Caharel, & Rossion, 2011; cf. Sauerland et al., 2017). The results for both response deadlines (i.e., 800 ms and 1500 ms) were analogous.

2 Error rates were analyzed with similar analyses. These results can be found in an online appendix on the open science framework (osf.io/4y7ge).

3 Participants recognized the thief (*M* = 0.85, SD = 0.36) and victim equally well (*M* = 0.90, SD = 0.30), *t*(81) = − 1.07, *p* = .288, *d* = − 0.12, 95% CI [− 0.31, 0.07]; as well as Actress A (*M* = 0.85, SD = 0.36) and B (*M* = 0.90, SD = 0.30), *t*(81) = 1.07, *p* = .288, *d* = − 0.12, 95% CI [− 0.31, 0.07].

4 A paired *t* test showed that participants identified the thief (*M* = 0.54, SD = 0.50) and victim with similar accuracy (*M* = 0.52, SD = 0.51), *t*(47) = 0.26, *p* = .799, *d* = 0.04, 95% CI [− 0.14, 0.19]; the same could be said for Actress A (*M* = 0.54, SD = 0.50) vs. B (*M* = 0.52, SD = 0.51), *t*(47) = 0.26, *p* = .799; *d* = 0.04, 95% CI [− 0.14, 0.19].
